# Supplementary material for: Recruitment of Mre11 to recombination sites during meiosis
Source: Nat Commun. 2026 Apr 7;17:4937. doi: 10.1038/s41467-026-71310-5 (PMC13233820; doi:10.1038/s41467-026-71310-5)
Supplement: Supplementary file 1 — Supplementary Information [file 41467_2026_71310_MOESM1_ESM.pdf]

Supplementary Information:

Recruitment of Mre11 to recombination sites during meiosis

Priyanka Priyadarshini[1,*], Mahesh Survi[1], Wael El Yazidi Mouloud[2,3], Regina Bohn[4], Steven Ballet[2], Neil Hunter[4,5], Alexander N. Volkov[3,6] and Corentin Claeys Bouuaert[1,*]

[1] Louvain Institute of Biomolecular Science and Technology, Université catholique de Louvain, 1348 Louvain-La-Neuve, Belgium.
[2] Research Group of Organic Chemistry, Vrije Universiteit Brussel (VUB), Pleinlaan 2, 1050 Brussels, Belgium
[3] Jean Jeener NMR Centre, Vrije Universiteit Brussel (VUB), Pleinlaan 2, 1050 Brussels, Belgium.
[4] HHMI, University of California, Davis, CA 95616, US.
[5] Department of Molecular & Cellular Biology, University of California, Davis, CA 95616, US.
[6] VIB-VUB Center for Structural Biology, VIB, Pleinlaan 2, 1050 Brussels, Belgium.

* Correspondence to priyanka.priyadarshini107@gmail.com, corentin.claeys@uclouvain.be

Supplementary Figures:

Supplementary Fig. 1: Properties of Mre11 nucleoprotein condensates.
Supplementary Fig. 2: Properties of MRX nucleoprotein condensates.
Supplementary Fig. 3: The C-terminal IDR of Mre11 is required for condensation.
Supplementary Fig. 4: Mre11 foci formation and expression during meiosis.
Supplementary Fig. 5: AlphaFold2 models of Mre11-Mer2 and conservation of Mre11-LLK and Mer2-EQEK residues.
Supplementary Fig. 6: Analysis of Mer2 – Mre11 interaction mutants.
Supplementary Fig. 7: The Mre11 C-terminus contains a SUMO-interaction motif.
Supplementary Fig. 8: NMR analysis of Smt3 bound to wild type and mutant SIM3 peptides.
Supplementary Fig. 9: ITC analysis of Smt3 binding to wild-type or mutant SIM3 peptides.
Supplementary Fig. 10: Protein expression and meiotic progression of Mre11 SIM3 mutants.

Supplementary Tables:

Supplementary Table 1. Oligonucleotides used in this study.
Supplementary Table 2. Plasmids used in this study.
Supplementary Table 3. Synthetic peptides used in this study.
Supplementary Table 4. Yeast strains used in this study.
Supplementary Table 5. Protein sequences used for AlphaFold modeling.
Supplementary Table 6. iPTM and TM scores of AlphaFold3-generated models of Mre11-C49-Mer2 complexes.

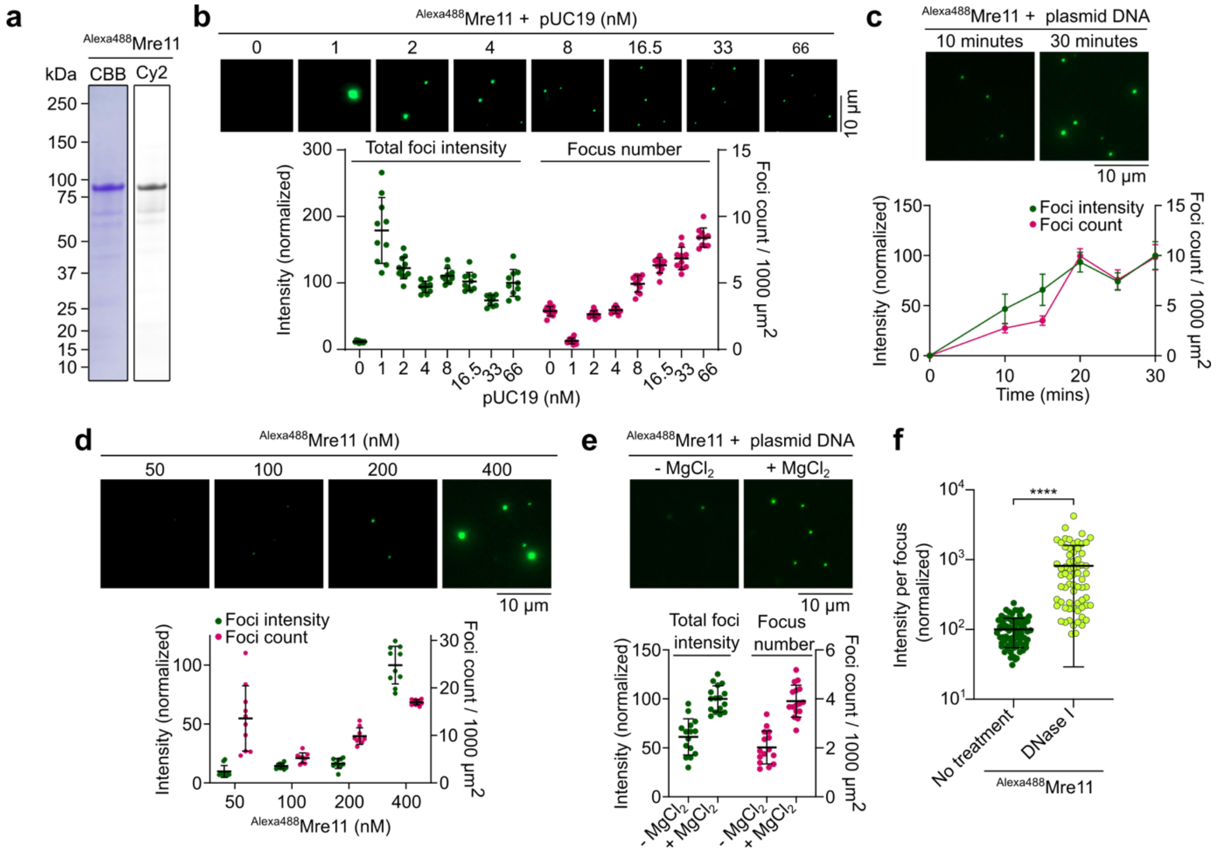

Supplementary Fig. 1: Properties of Mre11 nucleoprotein condensates.
a SDS-PAGE of Alexa488-labelled Mre11 stained with Coomassie Brilliant Blue (CBB) (left) and visualized with a Cy2 filter (right). **b** Effect of plasmid DNA (pUC19) concentration on 200 nM ^{Alexa488}Mre11 condensate, visualized by epifluorescence microscopy. Quantification shows total fluorescence intensity (green) in a field of view normalized to the highest DNA concentration, and total number of foci per 1000 μm^2 (magenta). n = 10 (0, 1, 4, 16.5, 33, 66 nM), n = 11 (2, 8 nM). Error bars represent mean ± SD from indicated (n) fields of view. **c** Time-dependent change in Mre11 condensate assembly. Reactions contained 400 nM ^{Alexa488}Mre11, 5.7 nM plasmid DNA and 5% PEG. Samples were collected at the indicated time points, immediately placed on a glass slide, covered with coverslip, and imaged. Foci intensities are normalized to the mean of the sample drawn at 30 minutes post-incubation. n = 15 (10 mins, 15 mins), n = 10 (20 mins, 25 mins, 30 mins). Error bars represent mean ± SD from indicated (n) fields of view. **d** Effect of Mre11 concentration on condensation in presence of plasmid substrate and 5% PEG. Foci intensities are normalized to the mean of the sample with 400 nM Mre11. Error bars represent mean ± SD from n = 10 fields of view. **e** Effect of presence of divalent cation (5 mM $MgCl_2$) (n = 16) on Mre11 condensates assembled in presence of pUC19. Foci intensities are normalized to reaction in the presence of magnesium. For experiment performed without magnesium (n = 15), 5 mM EDTA was included in the reaction. **f** Quantification of per focus intensity of Mre11 condensates treated with (n = 67) or without (n = 73) DNaseI as shown in Fig. 1b. Values on the y-axis are presented in log10 scale. Error bars are mean ± SD from indicated (n) foci.

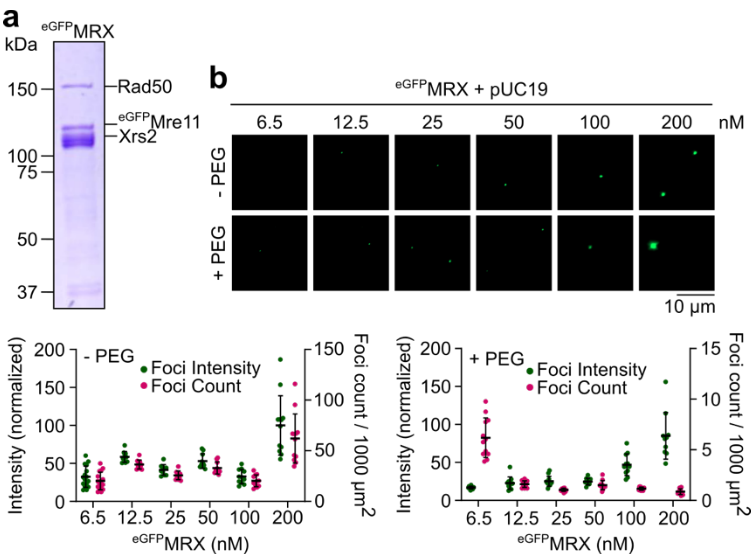

Supplementary Fig. 2: Properties of MRX nucleoprotein condensates.
a SDS-PAGE of eGFP-tagged Mre11-Rad50-Xrs2 (MRX) complex stained with Coomassie Brilliant Blue. Xrs2^{FLAG} appears higher in intensity than Mre11^{His} and Rad50 due to partial dissociation of MR subcomplex during affinity chromatography. **b** Effect of MRX concentration on nucleoprotein condensation in the presence or absence of 5% PEG. Foci intensities are normalized to the mean of the sample with 400 nM Mre11. For - PEG experiments: *n* = 16 (6.5 nM), *n* = 10 (12.5, 25, 50 nM), *n* = 11 (100 nM), *n* = 12 (200 nM) fields of view. For + PEG experiments: *n* = 12 (6.5 nM), *n* = 11 (12.5, 25, 50, 100 nM), *n* = 9 (200 nM) fields of view.

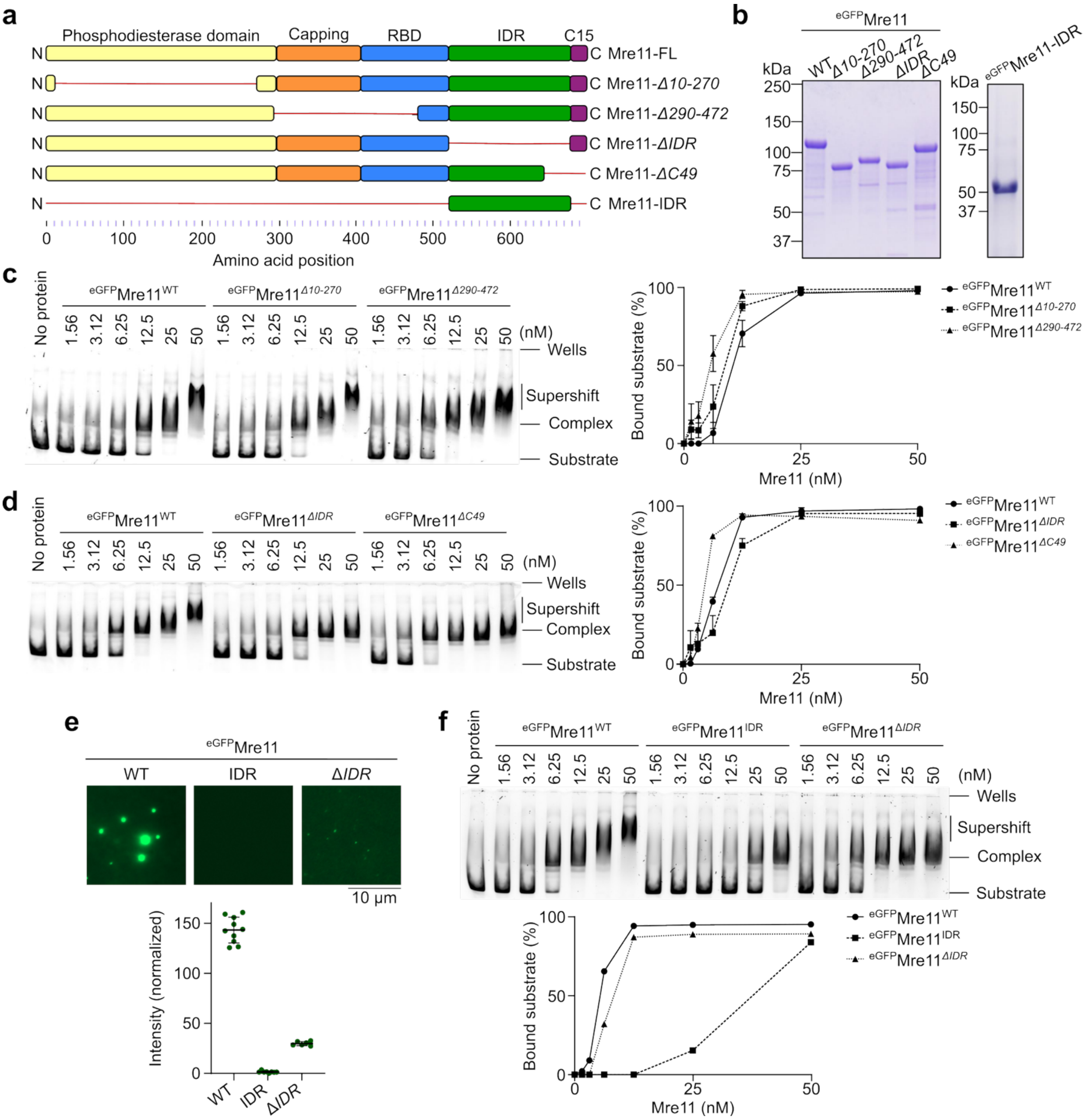

Supplementary Fig. 3: The C-terminal IDR of Mre11 is required for condensation.

a Cartoon diagram of full-length and truncated Mre11. RBD, Rad50-binding domain; IDR, intrinsically disordered region (residues 524-677). A red line indicates truncated regions. **b** SDS-PAGE of purified WT and truncated ^{eGFP}Mre11. **c, d** Effect of Mre11 truncations on plasmid DNA binding analyzed by gel shift assay. Error bars in (**c**) and (**d**) show ranges from n = 2 independent experiments. **e** *In vitro* condensation analysis of eGFP-tagged Mre11 (n = 16), Mre11-IDR (524-677) (n = 8), and Mre11-*ΔIDR* (*Δ524-677*) (n = 6). Foci intensities are normalized to the mean of wild-type ^{eGFP}Mre11. Error bars represent mean ± SD from indicated (n) fields of view. **f** Plasmid DNA binding of eGFP-tagged Mre11-IDR in comparison with Mre11and Mre11-*ΔIDR* analyzed by gel shift assay. In panels (**c**), (**d**), and (**f**), a well-defined band is observed, suggestive of a stoichiometric complex (Complex). With some constructs, species of lower electrophoretic mobility (supershift) are detected at high protein concentration, suggestive of higher-order oligomeric species. These depend on the Mre11 C-terminal IDR.

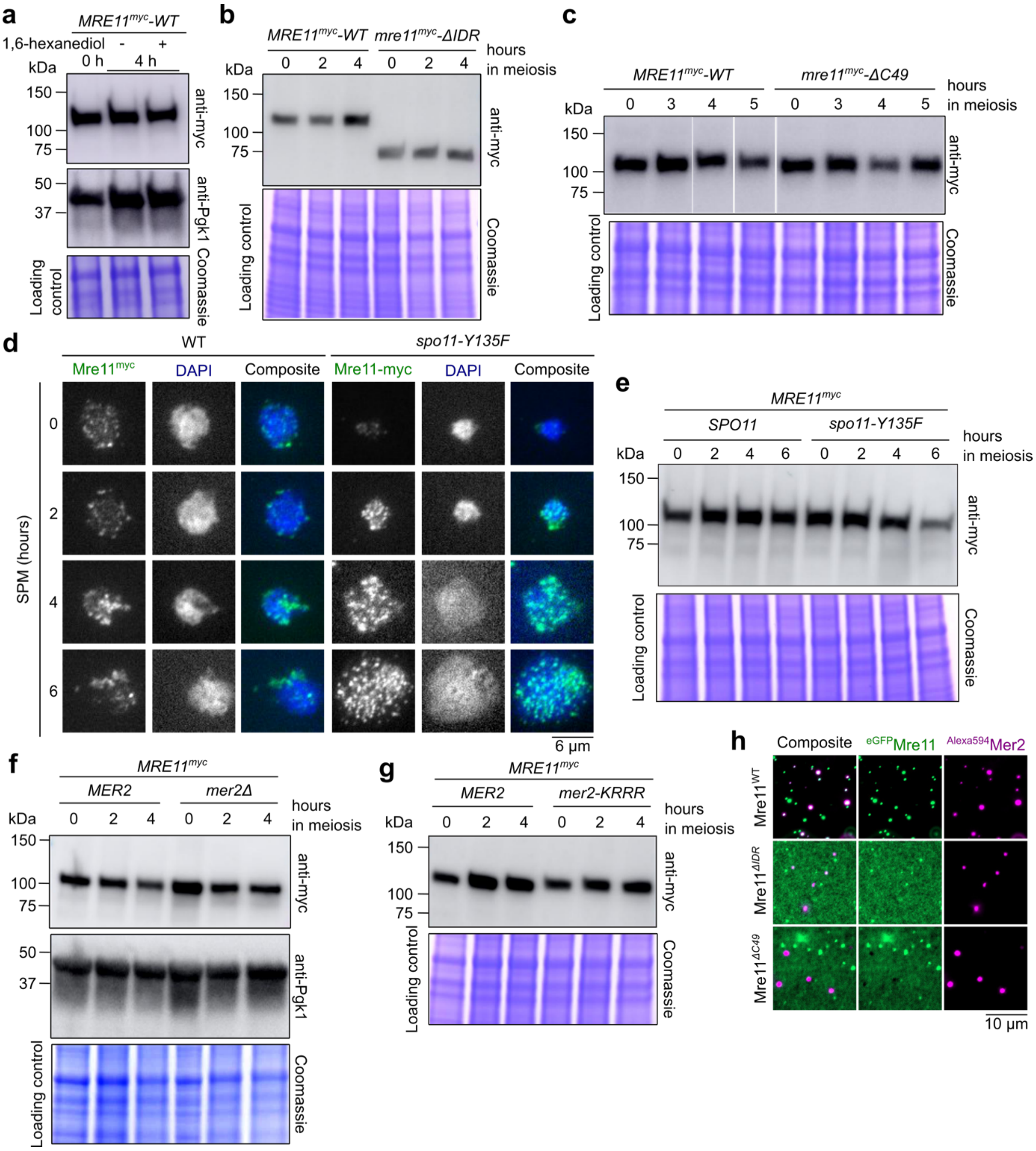

Supplementary Fig. 4: Mre11 foci formation and expression during meiosis.
(**a**–**c**, **e**–**g**) Western blot analysis of meiotic extracts of Mre11^{myc} in (**a**)1,6-hexanediol-treated Mre11^{myc} strains harvested 4 hours after transferring to SPM, (**b**) wild-type and Mre11-*ΔIDR* strains, (**c**) wild-type and Mre11-*ΔC49* strains, (**e**) wild-type and *spo11-Y135F* strains, (**f**) wild-type and *mer2Δ* strains, and (**g**) wild-type and *mer2-KRRR* strains. Coomassie-stained SDS-PAGE gels and anti-Pgk1 Western blots (panel 1) serve as loading controls. In panel (**c**), all samples were loaded on the same gel, but lanes were re-ordered. **d** Immunofluorescence on meiotic nuclear spreads of myc-tagged Mre11 in wild-type and *spo11-Y135F* strains. Contrary to previously published ChIP results showing similar association and dissociation kinetics of Mre11 in wild-type and *spo11-Y135F* backgrounds[41], in our hands Mre11 foci accumulate at late time points in a *spo11-Y135F* mutant. **h** Colocalization of fluorescently-labeled Mer2 with wild-type or truncated Mre11, as shown in Fig 4f. Here, the intensity of the green channel was not normalized between samples and the brightness was increased for the Mre11 truncations to visualize the background. This shows that, in addition to reduced condensation, the Mre11 truncations fail to co-localize with Mer2, in particular Mre11$^{\Delta C49}$.

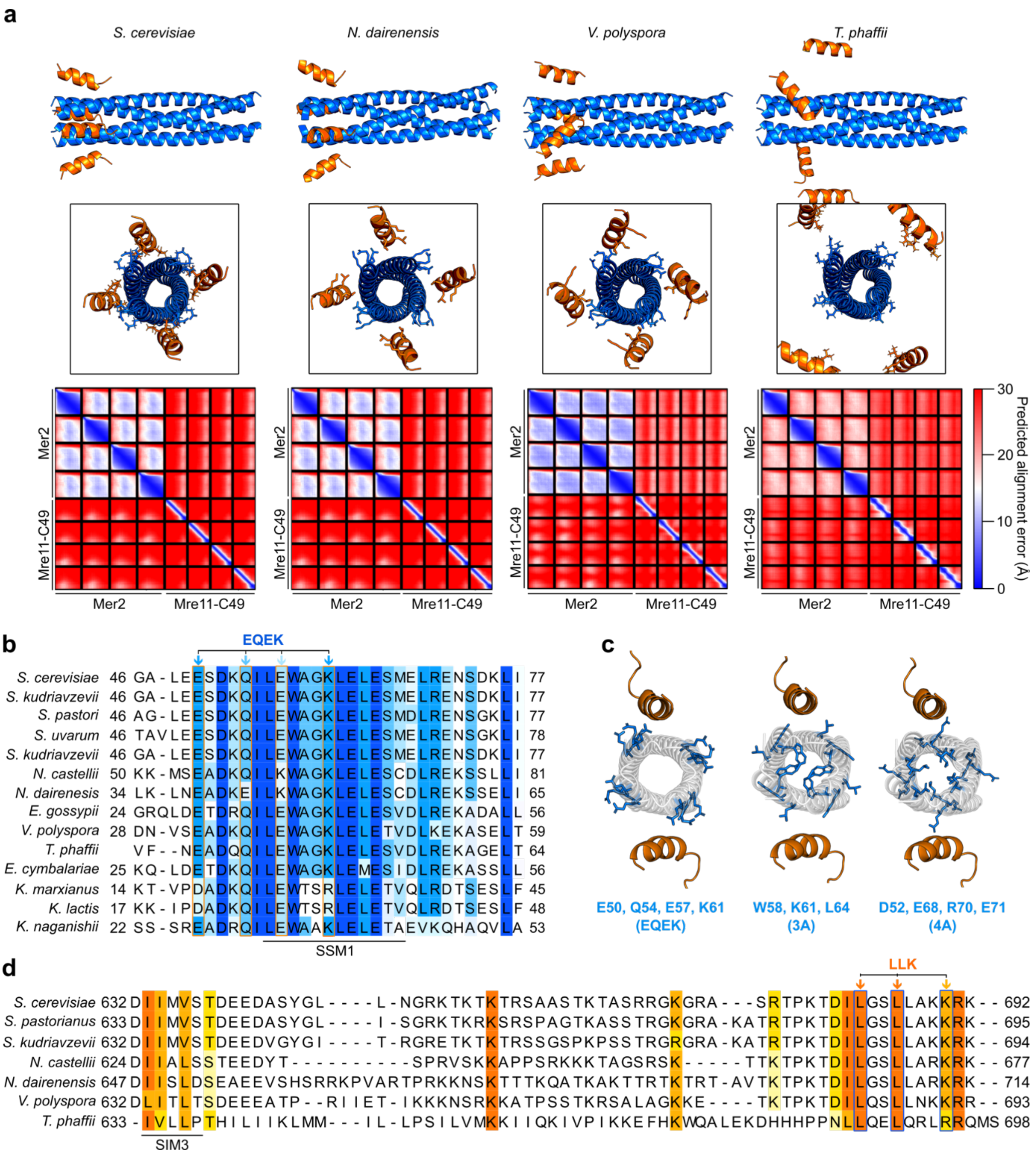

Supplementary Fig. 5: AlphaFold2 models of Mre11-Mer2 complexes and sequence conservation. **a** AlphaFold2 models of 4:4 Mre11-Mer2 interaction domains in various species of Saccharomycetaceae. Mre11 is shown in orange and Mer2 in blue. Mre11-LLK and Mer2-EQEK residues are shown as orange and blue sticks, respectively, in the lateral views. Disordered regions are omitted for clarity. All models are similar, except for *T. phaffi* that is aberrant. Predicted alignment error plots for each model is shown below. Dark blue represents low predicted error and high confidence, whereas lighter shades and red indicates low confidence, typical for flexible or disordered regions. NCBI accession numbers for Mer2 and Mre11, respectively, are as follows: *Saccharomyces cerevisiae* (CAA60944, BAA02017), *Naumovozyma dairenensis* (XP_003669210.1, XP_003672532.1), *Vanderwaltozyma polyspora* (XP_001647040.1, XP_001642997.1), and *Tetrapisispora phaffii* (XP_003683996.1, XP_003686402.1). The sequences used for AlphaFold modeling are provided in **Supplementary Table 5** and iPTM and pTM scores are provided in **Supplementary Table 6**. **b**–**d** Multiple sequence alignments of (**b**) Mer2 and (**d**) Mre11 in members of the Saccharomycetaceae family. EQEK residues are indicated by blue arrows and orange boxes and LLK residues are indicated by orange arrows and blue boxes. Alignment is colored based on percentage identity score on Jalview with a conservation threshold of 35% for Mer2 and 50% for Mre11. The previously-identified Mer2 signature sequence motif (SSM1) is indicated[46]. **c** Position of Mer2 EQEK residues (blue) on the AlphaFold2 model, and comparison with published 3A and 4A mutants[43]. Mre11 is in orange and Mer2 is in white.

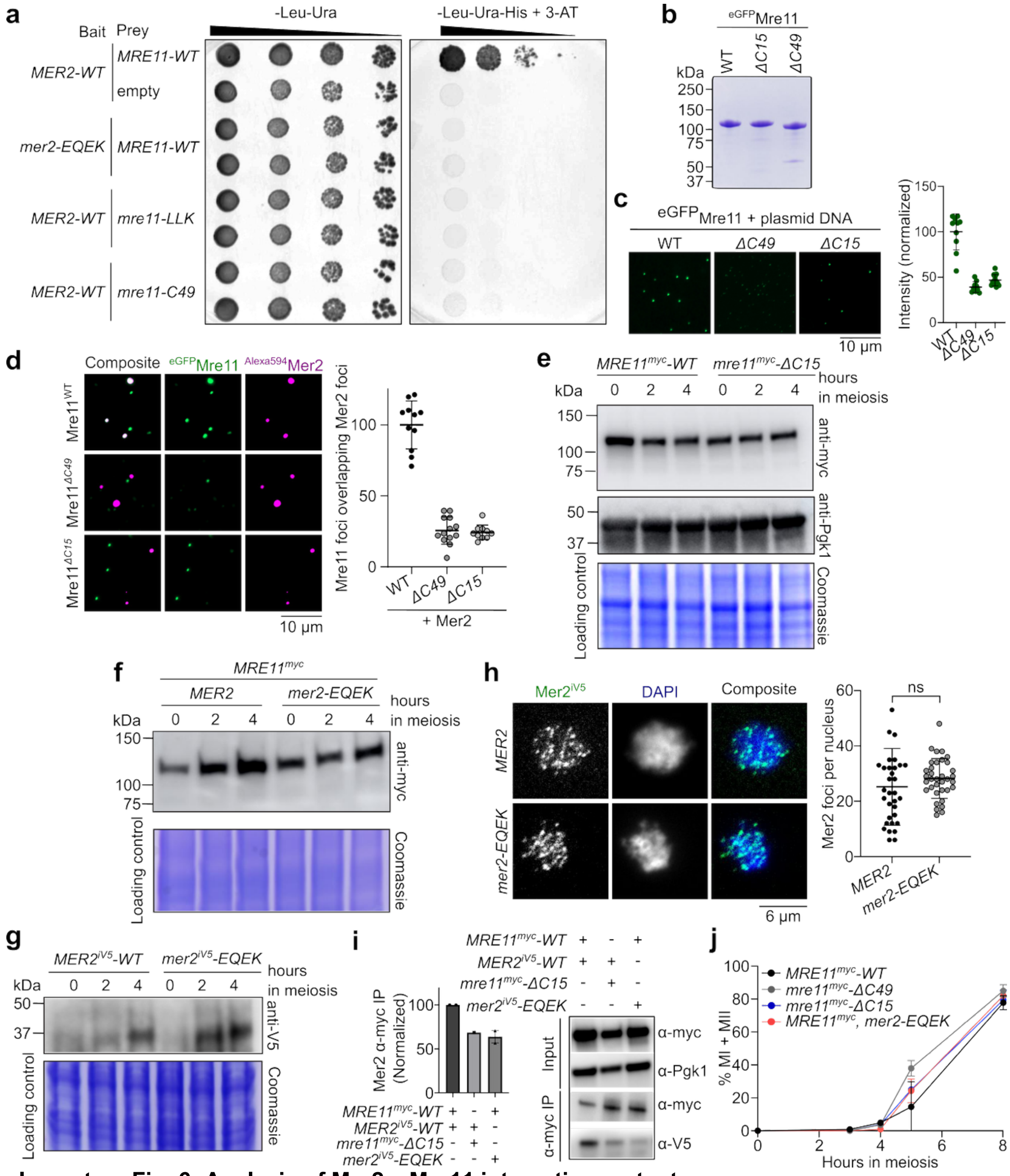

Supplementary Fig. 6: Analysis of Mer2 – Mre11 interaction mutants.

a Yeast-two-hybrid analysis between wild-type and mutant Mer2 and Mre11. **b** SDS-PAGE of purified WT and truncated ^{eGFP}Mre11. **c** *In vitro* condensation analysis of eGFP-tagged Mre11 (n = 10), Mre11-*ΔC49* (n = 9), and Mre11-*ΔC15* (n = 12). Foci intensities are normalized to the mean of wild-type ^{eGFP}Mre11. Error bars represent mean ± SD from indicated (n) fields of view. **d** Colocalization of fluorescently-labelled Mer2 with wild-type or truncated Mre11. Reactions containing 200 nM of ^{Alexa594}Mer2 and ^{eGFP}Mre11 were assembled separately for 10 minutes then mixed at 1:1 ratio for 30 minutes prior to imaging. Quantification shows the fraction of Mre11 foci overlapping Mer2 foci (white) in a field of view, normalized to Mre11-WT in (n = 6) for Mre11, (n = 13) for Mre11-*ΔC49*, and (n = 10). Mre11-*ΔC49*. Error bars represent mean ± SD from indicated (n) fields of view. **e**–**g** Western blot analysis of meiotic extracts of (**e**) *MRE11^{myc}-WT* and *mre11-ΔC15*^{myc}, (**f**) *MRE11^{myc}* in a *MER2* or *mer2-EQEK* strain, and (**g**) wild-type or mutant *MER2^{iV5}* strains. The *MER2^{iV5}* allele has an internal V5 tag between Mer2 amino acids 248 and 249. Coomassie-stained SDS-PAGE gels serve as loading controls. **h** Immunofluorescence on meiotic nuclear spreads of V5-tagged Mer2 in *WT* (n = 33) and *EQEK* strains (n = 35). **i** Co-immunoprecipitation assay between wild-type and mutant Mer2 and Mre11 and Mre11-ΔC15. Quantifications show mean and range from n = 2 experiments for Mre11-Mer2 and Mre11-*mer2-EQEK* and n = 1 for *mre11-ΔC15* and Mer2. **j** Meiotic progression of wild-type and mutant Mer2 and Mre11 strains (n = 2).

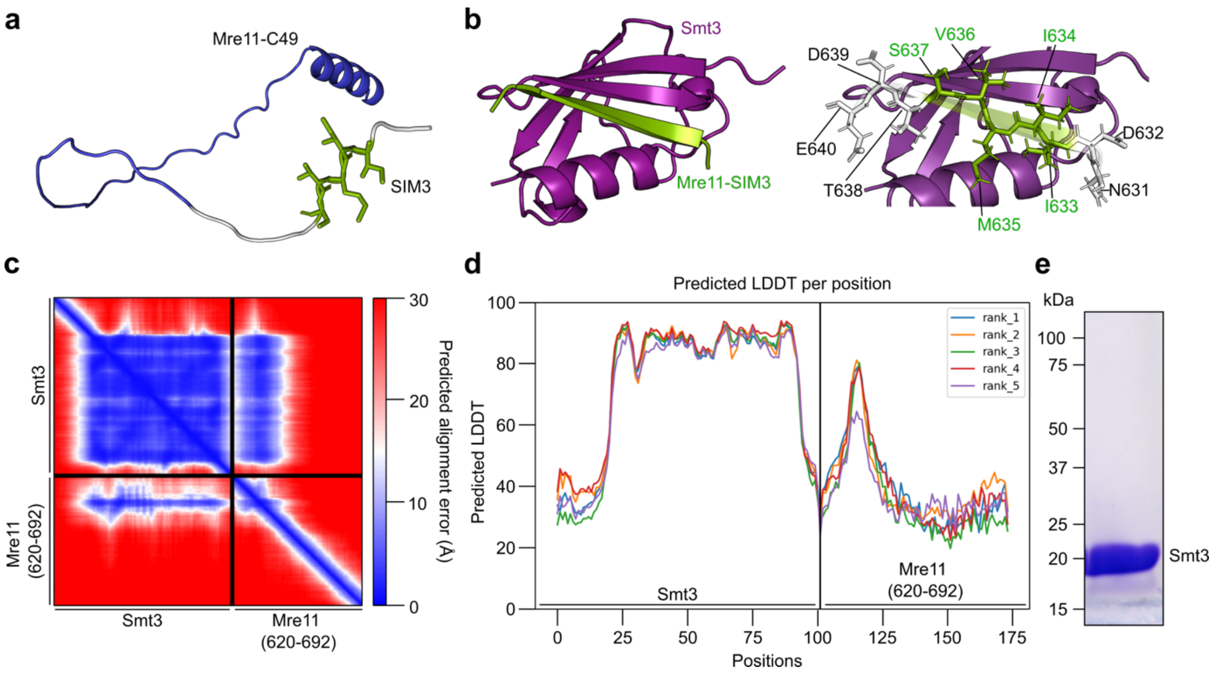

Supplementary Fig. 7: The Mre11 C-terminus contains a SUMO-interaction motif.
a Representation of Mre11 C-terminus (residues 630-692) from AlphaFold2 database (AF-P32829-F1-v4). The SIM3 motif (green) is located immediately before the Mre11-C49 residues (blue). **b** AlphaFold2 model of Smt3 (purple) and Mre11-SIM3 (residues 630-640) (green). Note that SIM3 is predicted to be disordered in panel a but folds as a β-sheet when bound to Smt3. Disordered regions of Smt3 and Mre11 are omitted for clarity. The sequences used for AlphaFold modeling are provided in **Supplementary Table 5**. **c** Predicted aligned error plot for AlphaFold2 model of Smt3 and Mre11-SIM3 (residues 620-692). The x- and y-axis represent amino acid residue positions. Darker blue represents low predicted error and high confidence, whereas lighter shades and red indicates low confidence, typical for flexible or disordered regions. **d** Predicted Local Distance Difference Test (pLDDT) plot for the AlphaFold2 model of Smt3 and Mre11-SIM3. The x-axis represents amino acid position and y-axis represents per-residue pLDDT score. Higher scores indicate greater confidence in local structure prediction. ipTM score of 0.69 and pTM score of 0.66 were obtained from AlphaFold2 prediction. **e** SDS-PAGE of purified His-tagged U-[^{13}C,^{15}N] Smt3.

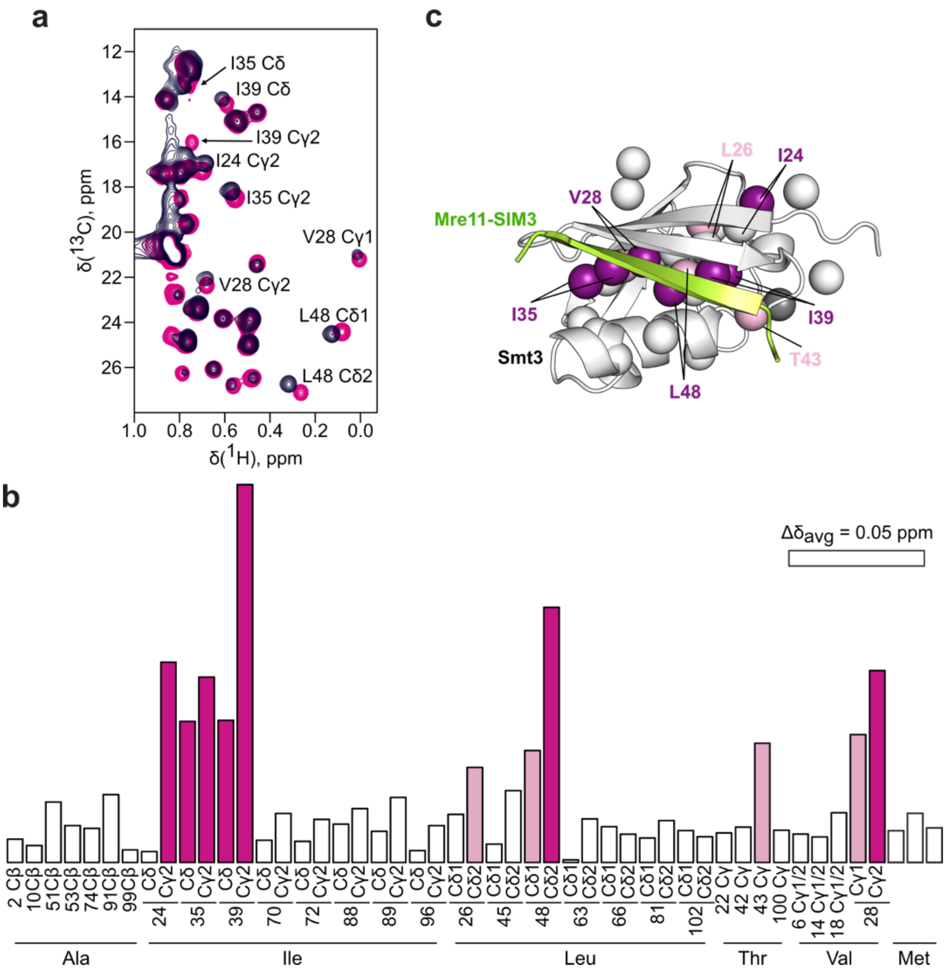

Supplementary Fig. 8: NMR analysis of Smt3 bound to wild type and mutant SIM3 peptides.
a Methyl regions of [^{1}H,^{13}C] HSQC spectra of the free Smt3 (black) and in the presence of 1.2 molar equivalents of wild type SIM3 peptide (magenta). The labels indicate the protein CH_3 groups showing the largest binding shifts. **b** Average methyl chemical shift perturbations ($\Delta\delta_{avg}$) of Smt3 upon binding to wild-type SIM3 peptide. The pink and magenta bars correspond to the CH_3 groups with $\Delta\delta_{avg} > 0.03$ ppm and > 0.05 ppm, respectively. For V6, V14, and V18, which show identical Cγ1 and Cγ2 NMR resonances, a single bar is shown. As the Cδ resonances of M49, M60, and M83 were not explicitly assigned in this work, the data for the methionine methyls are represented by unmarked bars. **c** Chemical shift mapping of the wild type SIM3 peptide binding. Smt3 methyls are shown as spheres, colored according to $\Delta\delta_{avg}$ as in panel b. The bound SIM3 peptide is in green, and the disordered Smt3 N- and C-termini are omitted for clarity.

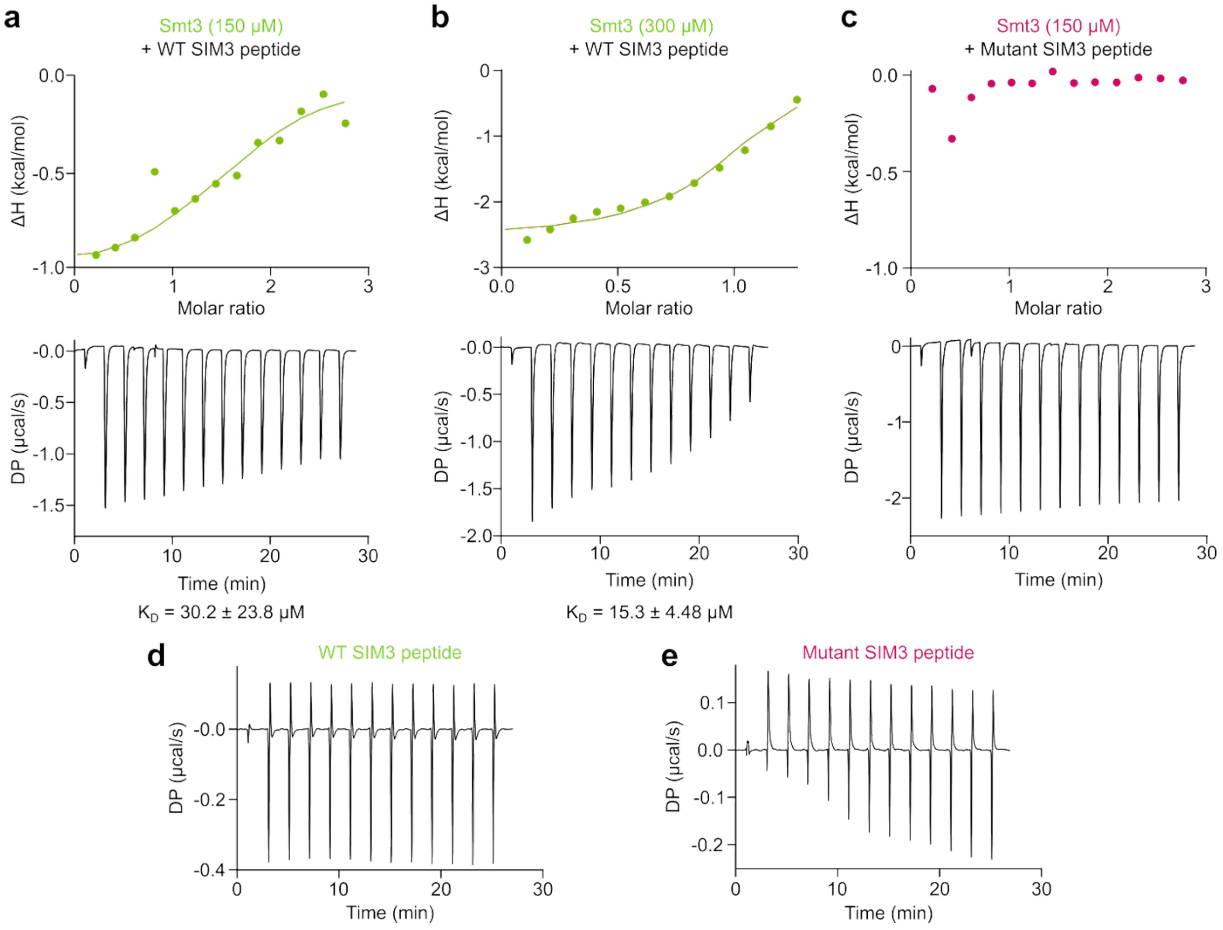

Supplementary Fig. 9: ITC analysis of Smt3 binding to wild-type or mutant SIM3 peptides.
a–**c** Top panels, integrated heat peaks ΔH (kcal/mol) as a function of molar ratio (peptide/protein concentration) after buffer subtraction and offset correction. Bottom panels, raw data plots indicating differential power (µcal/s) after baseline correction as a function of time. The data was fitted to a single binding site model and the number of binding sites (N) was set to 1. The model allowed for the calculation of the equilibrium dissociation constant K_D as provided below each graph. **d, e** Integrated heat peaks ΔH (kcal/mol) as a function of molar ratio (peptide/protein concentration) after buffer subtraction and offset correction performed on peptide injections into buffer (control).

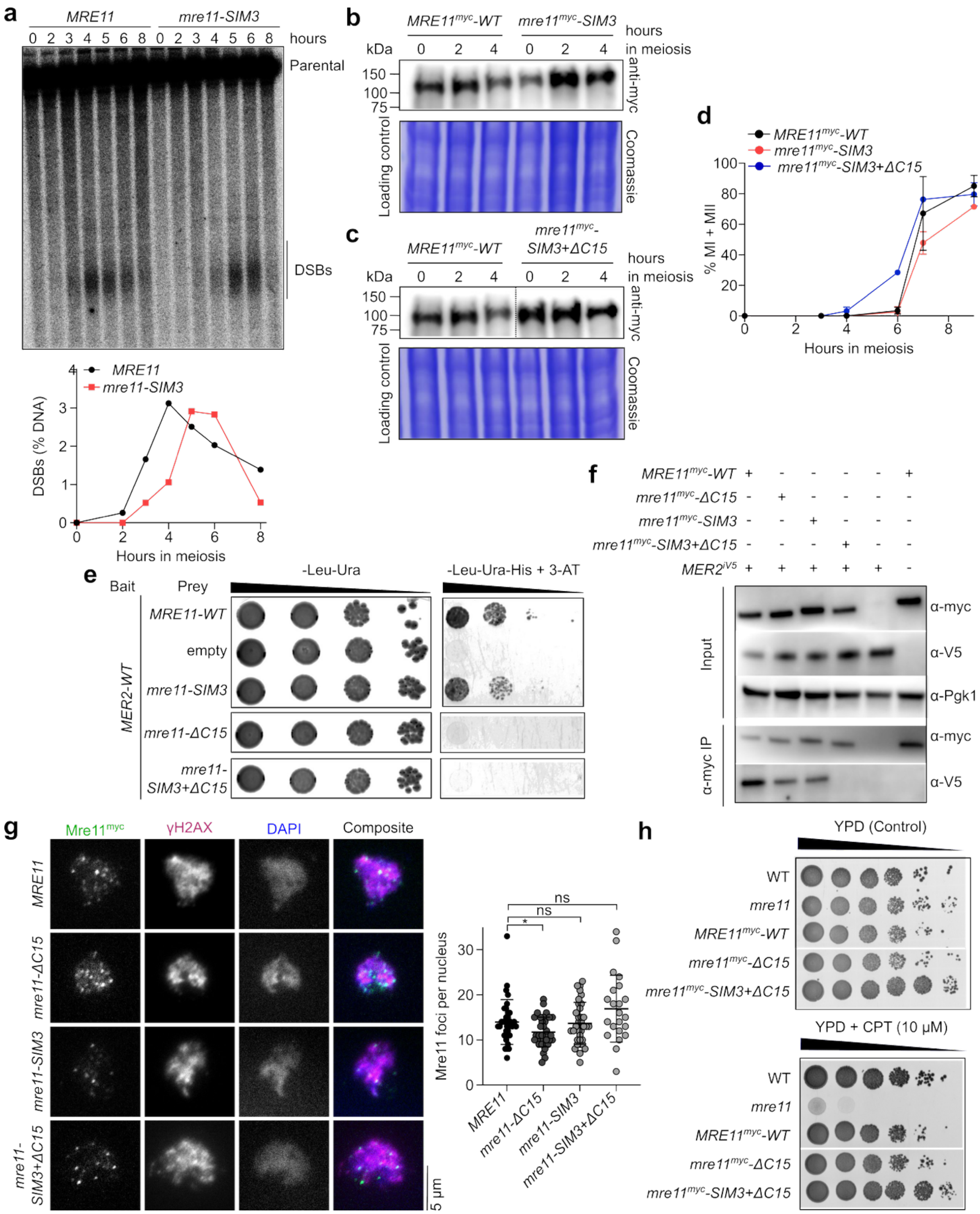

Supplementary Fig. 10: Protein expression and meiotic progression of Mre11 SIM3 mutants.
a Southern blot analysis of meiotic DSB formation at the *GAT1* hotspot. **b, c** Western blot analysis of meiotic extracts of (**b**) *MRE11*^{myc}*-WT* and *mre11-SIM3*^{myc}, (**c**) *MRE11*^{myc}*-WT* and *mre11-SIM3+ΔC15*^{myc}. Coomassie-stained SDS-PAGE gels serve as loading controls. **d** Meiotic progression, as indicated by the percentage of cells that have undergone the first or second meiotic divisions (MI + MII) (n = 2). **e** Yeast-two-hybrid analysis between Mer2 wild-type (binding domain, bait) and Mre11 wild-type and mutants (activation domain, prey). **f** Co-immunoprecipitation assay between Mer2 and Mre11 mutants. **g** Effect of Mre11^{myc} truncations on MMS-induced foci formation as visualized by immunofluorescence. Error bars show mean ± SD of Mre11^{myc} foci for *WT* (n = 34), *mre11-ΔC15* (n = 22), *mre11-SIM3* (n = 34) and *mre11-SIM3+ΔC15* (n = 34). Two-tailed unpaired t-tests were performed between *MRE11* and *mre11-ΔC15* (p = 0.032), *mre11-SIM3* (p = 0.802) and *mre11-SIM3+ΔC15* (p = 0.081). **h** Sensitivity of wild-type and truncated Mre11^{myc} strains to camptothecin (CPT). Ten-fold serial dilutions from saturated cultures are shown, with dilutions on YPD plates as control.

Primer No.	Sequence
cb1351	CCGTCCCACCATCGGGCGCGGATCCACCATGGACTATCCTGATCC
cb1352	GCTCGTCGACGTAGGCCTTTGAATTCCTAATGATGGTGGTGGTGATGATGATGGTGGTGTTTTCTT TTCTTAGCAAGGAGAC
cb1353	CCGTCCCACCATCGGGCGCGGATCCACCATGAGCGCTATCTATAAATTATCTATTCAG
cb1354	GCTCGTCGACGTAGGCCTTTGAATTCTCAATAAGTGACTCTGTTAATATCG
cb1355	CCGTCCCACCATCGGGCGCGGATCCACCATGTGGGTAGTACGATACC
cb1356	GCTCGTCGACGTAGGCCTTTGAATTCTCATTTGTCATCATCGTCCTTGTAATCCTTGTCATCGTCAT CTTTATAATCTCCTTTTCTTCTTTTGAACG
cb1424	CGACTCACTATAGGGCGAATTGGAGCTCGAAAATAAAGGCATCTACAAATCTC
cb1425	CCTCACTAAAGGGAACAAAAGCTGGGTACCTTCAGCAAATAGCATTCAAC
cb1435	AGCGTCCTCTTCGTC
cb1458	CACCACCATCATCATCACCACCACCATCATTAGGAATTCAAAGGCCTAC
cb1486	GGATTGGAAGTACAGGTTTTC
cb1559	GAAAACCTGTACTTCCAATCCAGTTATGGTTTACTTAATGGTC
cb1561	TATCGCCTTGTCCGATGCCTCTAAAGCGCCTGC
cb1562	CTGGCATGGGCAGGTGCACTGGAATTGGAGAGTATG
dam005	CCCTGCTGGGCCTGGACAGCACCCGTAAGAAGTCCGTGCC
dam006	GGTTGGGGATGGGCTTGCCCTGCTTATCGTGGGCT
pp3	ACTGACGAAGAGGACGC
pp4	AGCAGCTGCAGCTGCGTCATTCTCATCAATATC
pp24	AGCACGCTTAACCTGTTTTATAAGC
pp25	CCAAAGACGGATATTCTTGG
pp26	TATTGTGTCTGGATCAGGAT
pp27	GAGGCTGAGGCACAACCCAA
pp28	TGCTTCTCCATACTTTATGTC
pp29	GTAGATAAAGATGAGAAAACAGCTC
pp46	GAACAAAAGCTGGGT
pp55	CACCACCATCATCATCACCACCACC
pp59	GGCATGGACGAGCTGTACAAGGGATCCACCATGAACAGTGTTAGGCCGACTCC
pp60	GGTGGTGGTGATGATGATGGTGGTGCTTTGGCGTCCTTGATGCTCTTCC
pp61	GGTGGATCCCTTGTACAGCTCGTCCATGCC
pp72	GAACAAAAGCTGGGTACCGG
pp73	CGTCCTTGATGCTCTTCCTTT
pp74	CACCACCATCATCATCACCA
pp84	CTAAGGCCAGAAAAATTCGAGCTCGGCGCGCC
pp85	CAAGAGCACTTCCCGCAATATCCGTCTTTGGCG
pp92	AGTTATGGTTTACTTAATGGT
pp120	CTCGAGCACCACCACCA
pp127	GGTATATCTCCTTCTTAAAG
pp132	CTTTAAGAAGGAGATATACCATGAAAATCGAAGAAGGTAA
pp135	TGGTGGTGGTGGTGCTCGAGTCAGGATTGGAAGTACAGGTTTTC
pp136	TGGTGGTGGTGGTGCTCGAGTCATTTTCTTTTCTTAGCAAGGA
pp148	CCATTAAGTAAACCATAACTCTTGTACAACTCATCCATAC
pp149	AAAACCTGTACTTCCAATCCATGGTGTCTAAAGGTGAAGC
RB70	AGCTGCGTCATTCTCATCAATATCAACATCATCTTG
RB267	GCAGCCGCTACTGACGAAGAGGACGCTAGTTATGG
RB268	TAAAAGCTGTCGAGAAGTACTAG
RB269	CGTCCTTGATGCTCTTCC
RB308	CGTCTTTGGCGTCCTTGATGCTC
RB309	AGTTATGGTTTACTTAATGGTCGAAAAACAAAAAC
RB310	GGATCCGGGCCCCTGG
RB311	CTGGCATGGGCAGGTGCACTGGAATTGG
RB312	TATAGCCTTGTCCGATGCCTCTAAAGCG
RB313	GATATTGCTGGAAGTGCTCTTGCTAAGGCTAGAAAATAA

Supplementary Table 1. Oligonucleotides used in this study.

Description	Plasmid	Reference
SUMO-Mer2 in pSMT3	pCCB750	9
SUMO-eGFP-Mer2 in pSMT3	pCCB777	9
mScarlet-Mer2 in pSMT3	pCCB785	This study
Mre11-10xHis in pFastBac1	pCCB865	This study
Rad50 in pFastBac1	pCCB866	This study
Xrs2-2xFLAG in pFastBac1	pCCB867	This study
eGFP-Mre11-10xHis in pFastBac1	pCCB942	This study
eGFP-Mre11-*ΔC49*-10xHis in pFastBac1	pCCB943	This study
Smt3-6xHis in pET28b	pCCB998	73
MBP-Mre11-C49 + HisSUMO-Mer2 in pETDuet1	pCCB1040	This study
Mer2-*EQEK*::HphMX in PCR-Blunt II-TOPO	pCCB1046	This study
Mer2^{iV5}-*EQEK*::HphMX in PCR-Blunt II-TOPO	pCCB1048	This study
Mre11-8xmyc::URA3 in PCR-Blunt II-TOPO	pry2	This study
eGFP-Mre11-*Δ290-472*-10xHis in pFastBac1	pry5	This study
eGFP-Mre11-*ΔIDR*-10xHis in pFastBac1	pry6	This study
eGFP-Mre11-*Δ10-270*-10xHis in pFastBac1	pry7	This study
Mre11-*ΔC49*-8xmyc::URA3 in PCR-Blunt II-TOPO	pry24	This study
Mre11-*ΔIDR*-8xmyc::URA3 in PCR-Blunt II-TOPO	pry30	This study
eGFP-Mre11-IDR-10xHis in pFastBac1	pry41	This study
Mre11-*ΔC15*-8xmyc::URA3 in PCR-Blunt II-TOPO	pry42	This study
eGFP-Mre11-*ΔC15*-10xHis in pFastBac1	pry44	This study
Mre11-*SIM3*-8xmyc::URA3 in PCR-Blunt II-TOPO	pry56	This study
Mre11-*SIM3+ΔC15*-8xmyc::URA3 in PCR-Blunt II-TOPO	pry57	This study
MBP-Mre11-C49 + HisSUMO-Mer2-*EQEK* in pETDuet1	pry59	This study
MBP-Mre11-C49-*LLK* + HisSUMO-Mer2 in pETDuet1	pry61	This study
MBP in pET28a	pry94	This study
MBP-Mre11-C49 in pET28a	pry99	This study
MBP-mScarlet-Mre11-C49 in pET28a	pry109	This study
Mer2^{iV5}::HpHMX in PCR-Blunt II-TOPO	pDAM003	This study
Mer2::HphMX in PCR-Blunt II-TOPO	pMH002	45
HisSUMO-Mer2 coiled-coil in pET28a	pDD065	45
Mer2 in pGBDU-C1	pWL1592	43
Mre11 in pGAD-C1	pWL1596	43
pGAD-C1	pWL1565	43
Mre11-*SIM3* in pGAD-C1	pNH1371	This study
Mre11-*ΔC15* in pGAD-C1	pNH1372	This study
Mre11-*SIM3+ΔC15* in pGAD-C1	pNH1373	This study
Mer2-EQEK (AAAA) in pGBDU-C1	pNH1422	This study
Mre11-LLK (AAA) in pGAD-C1	pNH1423	This study
Mre11-C49 in pGAD-C1	pNH1424	This study

Supplementary Table 2. Plasmids used in this study.

Name	Sequence	Brutoformula	Calculated mass [M+H]$^+$	Measured mass [M+H]$^+$
Mre11-SIM - WT	ENDIIMVSTDE	$C_{51}H_{84}N_{12}O_{23}S$	1265.5571	1265.5593
Mre11-SIM - mutant	ENDAIAVSTDE	$C_{46}H_{74}N_{12}O_{23}$	1163.5068	1163.5074

Supplementary Table 3. Synthetic peptides used in this study.

Strain	Genotype	Reference
CBY6	*MATa, ho::LYS2, lys2, ura3, leu2::hisG, trp1::hisG*	
CBY7	*MATα, ho::LYS2, lys2, ura3, leu2::hisG, trp1::hisG*	
CBY29	*MATα, ho::LYS2, lys2, ura3, leu2::hisG, mer2Δ::KanMX4*	
CBY317	*MATα, ho::LYS2, lys2, ura3, leu2, arg4, SPO11-Y135F-His6-flag3-loxP-hphMX-loxP*	
CBY375	*MATa, ho::LYS, ura3, leu2::hisG, trp1::hisG, MRE11-myc::URA3, arg4-nsp*	
CBY596	*MATa, ho::LYS2, lys2, ura3, leu2::hisG, trp1::hisG, MRE11-8xmyc::URA3*	This study
CBY597	*MATα, ho::LYS2, lys2, ura3, leu2::hisG, trp1::hisG, MRE11-8xmyc::URA3*	This study
CBY613	*MATα, ho::LYS2, lys2, ura3, leu2::hisG, trp1::hisG, mer2-KRRR::hphMX4*	45
CBY727	*MATa, ho::LYS2, lys2, ura3, leu2::hisG, trp1::hisG, arg4, MRE11-8xmyc::URA3, SPO11-Y135F-His6-flag3-loxP-hphMX-loxP*	This study
CBY728	*MATα, ho::LYS2, lys2, ura3, leu2::hisG, trp1::hisG, arg4, MRE11-8xmyc::URA3, SPO11-Y135F-His6-flag3-loxP-hphMX-loxP*	This study
CBY845	*MATa, ho::LYS2, lys2, ura3, leu2::hisG, trp1::hisG, mer2-KRRR::hphMX4, MRE11-8xmyc::URA3*	This study
CBY846	*MATα, ho::LYS2, lys2, ura3, leu2::hisG, trp1::hisG, mer2-KRRR::hphMX4, MRE11-8xmyc::URA3*	This study
CBY849	*MATa, ho::LYS2, lys2, ura3, leu2::hisG, trp1::hisG, mer2Δ::MER2^{iV5}::hphMX, MRE11-8xmyc::URA3*	This study
CBY850	*MATα, ho::LYS2, lys2, ura3, leu2::hisG, trp1::hisG, mer2Δ::MER2^{iV5}::hphMX, MRE11-8xmyc::URA3*	This study
CBY864	*MATα, ho::LYS2, lys2, ura3, leu2::hisG, trp1::hisG, mer2Δ::MER2^{iV5}::hphMX*	This study
CBY868	*MATa, ho::LYS2, lys2, ura3, leu2::hisG, trp1::hisG, mer2Δ::MER2^{iV5}::hphMX*	This study
CBY872	*MATa, ho::LYS2, lys2, ura3, leu2::hisG, trp1::hisG, mre11-ΔC49-8xmyc::URA3*	This study
CBY873	*MATα, ho::LYS2, lys2, ura3, leu2::hisG, trp1::hisG, mre11-ΔC49-8xmyc::URA3*	This study
CBY1013	*MATa, ho::LYS2, lys2, ura3, leu2::hisG, trp1::hisG, mre11-ΔIDR-8xmyc::URA3*	This study
CBY1014	*MATα, ho::LYS2, lys2, ura3, leu2::hisG, trp1::hisG, mre11-ΔIDR-8xmyc::URA3*	This study
CBY1017	*MATa, ho::LYS2, lys2, ura3, leu2::hisG, trp1::hisG, mer2-EQEK::hphMX4*	This study
CBY1018	*MATα, ho::LYS2, lys2, ura3, leu2::hisG, trp1::hisG, mer2-EQEK::hphMX4*	This study
CBY1021	*MATa, ho::LYS2, lys2, ura3, leu2::hisG, trp1::hisG, mer2^{iV5}-EQEK::hphMX4*	This study
CBY1022	*MATα, ho::LYS2, lys2, ura3, leu2::hisG, trp1::hisG, mer2^{iV5}-EQEK::hphMX4*	This study
CBY1025	*MATa, ho::LYS2, lys2, ura3, leu2::hisG, trp1::hisG, MRE11-8xmyc::URA3, mer2Δ::KanMX4*	This study
CBY1026	*MATα, ho::LYS2, lys2, ura3, leu2::hisG, trp1::hisG, MRE11-8xmyc::URA3, mer2Δ::KanMX4*	This study
CBY1027	*MATa, ho::LYS2, lys2, ura3, leu2::hisG, trp1::hisG, mre11-ΔC15-8xmyc::URA3*	This study
CBY1028	*MATα, ho::LYS2, lys2, ura3, leu2::hisG, trp1::hisG, mre11-ΔC15-8xmyc::URA3*	This study
CBY1034	*MATa, ho::LYS2, lys2, ura3, leu2::hisG, trp1::hisG, mer2-EQEK::hphMX4, MRE11-8xmyc::URA*	This study
CBY1035	*MATα, ho::LYS2, lys2, ura3, leu2::hisG, trp1::hisG, mer2-EQEK::hphMX4, MRE11-8xmyc::URA3*	This study
CBY1048	*MATa, ho::LYS2, lys2, ura3, leu2::hisG, trp1::hisG, mre11-SIM3+ΔC15-8xmyc::URA3*	This study
CBY1049	*MATα, ho::LYS2, lys2, ura3, leu2::hisG, trp1::hisG, mre11-SIM3+ΔC15-8xmyc::URA3*	This study
CBY1237	*MATa, ho::LYS2, lys2, ura3, leu2::hisG, trp1::hisG, mre11-SIM3-8xmyc::URA3*	This study
CBY1238	*MATα, ho::LYS2, lys2, ura3, leu2::hisG, trp1::hisG, mre11-SIM3-8xmyc::URA3*	This study
yWL365	*MATa, ura3-52, leu2-3, his3, trp1, gal4del, gal80del, GAL2-ADE2, LYS2::GAL1-HIS3, met2::GAL7-lacZ*	43

Supplementary Table 4. Yeast strains used in this study.

Protein	Organism	NCBI Accession No.	Input sequence
Mer2	*Saccharomyces cerevisiae*	CAA60944	KVTNAGALEESDKQILEWAGKLELESMELRENSDKLIKVLNENSKTLCKSLNKFNQLLEQ
	Naumovozyma dairenensis	XP_003669210.1	SSPLKLNEADKEILKWAGKLELESCDLREKSSELIGLLKKNSTQLTSVISTLNEIVIST
	Tetrapisispora phaffii	XP_003683996.1	PVKETVFNEADQQILEWAGKLELESVDLREKAGELTTVLKRNSDRLYSVMEQLNKNLKN
	Vanderwaltozyma polyspora	XP_001647040.1	SGRDNVSEADKQILEWAGKLELETVDLKEKASELTNVFNESNTKLLELVARLNDHLGS
Mre11	*Saccharomyces cerevisiae*	BAA02017	SYGLLNGRKTKTKTRSAASTKTASRRGKGRASRTPKTDILGSLLAKKRK
	Naumovozyma dairenensis	XP_003672532.1	VARTPRKKNSKTTTKQATKAKTTRTKTRTAVTKTPKTDILGSLLARKRK
	Tetrapisispora phaffii	XP_003686402.1	ILLPSILVMKKIIQKIVPIKKEFHKWQALEKDHHHPPNLLQELQRLRRQMS
	Vanderwaltozyma polyspora	XP_001642997.1	ATPRIIETIKKKNSRKKATPSSTKRSALAGKKETKTPKTDILQSLLNKKRR
Smt3	*Saccharomyces cerevisiae*	WNF20333	MSDSEVNQEAKPEVKPEVKPETHINLKVSDGSSEIFFKIKKTTPLRRLMEAFAKRQGKEMDSLRFLYDGIRIQADQTPEDLDMEDNDIIEAHREQIGGATY
Mre11-SIM3	*Saccharomyces cerevisiae*	BAA02017 (Mre11 (residues 620-692)	NDAQDDVDIDENDIIMVSTDEEDASYGLLNGRKTKTKTRSAASTKTASRRGKGRASRTPKTDILGSLLAKKRK

Supplementary Table 5. Protein sequences used for AlphaFold modeling.

Organism	iPTM score	pTM score
Saccharomyces cerevisiae	0.33	0.5
Naumovozyma dairenensis	0.38	0.44
Tetrapisispora phaffii	0.19	0.27
Vanderwaltozyma polyspora	0.33	0.47

Supplementary Table 6. iPTM and pTM scores of AlphaFold2-generated models of Mre11-C49-Mer2 complexes.
