# Supplementary material for: Recruitment of Mre11 to recombination sites during meiosis
Source: Nat Commun. 2026 Apr 7;17:4937. doi: 10.1038/s41467-026-71310-5 (PMC13233820; doi:10.1038/s41467-026-71310-5)
Supplement: Supplementary file 2 — Reporting Summary [file 41467_2026_71310_MOESM2_ESM.pdf]

nature portfolio

Corresponding author(s): Priyanka Priyadarshini, Corentin Claeys Bouuaert

Last updated by author(s): 2026/02/26

# Reporting Summary

Nature Portfolio wishes to improve the reproducibility of the work that we publish. This form provides structure for consistency and transparency in reporting. For further information on Nature Portfolio policies, see our Editorial Policies and the Editorial Policy Checklist.

## Statistics

For all statistical analyses, confirm that the following items are present in the figure legend, table legend, main text, or Methods section.

| n/a | Confirmed | |
|---|---|---|
| ☐ | ☒ | The exact sample size (*n*) for each experimental group/condition, given as a discrete number and unit of measurement |
| ☐ | ☒ | A statement on whether measurements were taken from distinct samples or whether the same sample was measured repeatedly |
| ☐ | ☒ | The statistical test(s) used AND whether they are one- or two-sided<br>*Only common tests should be described solely by name; describe more complex techniques in the Methods section.* |
| ☒ | ☐ | A description of all covariates tested |
| ☐ | ☒ | A description of any assumptions or corrections, such as tests of normality and adjustment for multiple comparisons |
| ☐ | ☒ | A full description of the statistical parameters including central tendency (e.g. means) or other basic estimates (e.g. regression coefficient) AND variation (e.g. standard deviation) or associated estimates of uncertainty (e.g. confidence intervals) |
| ☐ | ☒ | For null hypothesis testing, the test statistic (e.g. *F*, *t*, *r*) with confidence intervals, effect sizes, degrees of freedom and *P* value noted<br>*Give P values as exact values whenever suitable.* |
| ☒ | ☐ | For Bayesian analysis, information on the choice of priors and Markov chain Monte Carlo settings |
| ☒ | ☐ | For hierarchical and complex designs, identification of the appropriate level for tests and full reporting of outcomes |
| ☒ | ☐ | Estimates of effect sizes (e.g. Cohen's *d*, Pearson's *r*), indicating how they were calculated |

*Our web collection on statistics for biologists contains articles on many of the points above.*

## Software and code

Policy information about availability of computer code

| | |
|---|---|
| Data collection | Microscopy data was acquired with ZEN 2.3 pro (ZEISS), EMSA gels and Southern blots were imaged with Amersham Typhoon control software (Cytiva), and SDS-PAGE gels were imaged with Amersham Imager 600 1.2.0. NMR spectra was collected with Bruker Avance III HD 800 MHz spectrometer. |
| Data analysis | Gels and blots were quantified using ImageJ 1.53t and plotted using GraphPad Prism (version 10). Structure predictions were made with AlphaFold3. Microscopy images of condensation assays were analyzed in ImageJ 1.53t using a custom-made script available at https://github.com/claeysbouuaert/scripts (Claeys Bouuaert et al. 2021) and described in Materials and Methods. NMR data were processed in TopSpin 3.6 (Bruker) or NMRPipe and analyzed in CCPNMR. ITC data was fit using Microcal LLC ITC200 Origin software. |

For manuscripts utilizing custom algorithms or software that are central to the research but not yet described in published literature, software must be made available to editors and reviewers. We strongly encourage code deposition in a community repository (e.g. GitHub). See the Nature Portfolio guidelines for submitting code & software for further information.

# Data

Policy information about availability of data

All manuscripts must include a data availability statement. This statement should provide the following information, where applicable:

- Accession codes, unique identifiers, or web links for publicly available datasets
- A description of any restrictions on data availability
- For clinical datasets or third party data, please ensure that the statement adheres to our policy

The assigned 1H, 13C, and 15N chemical shifts of the free and bound Smt3 have been deposited in the Biological Magnetic Resonance Bank (http://www.bmrb.wisc.edu/) under the accession number 53209.

# Research involving human participants, their data, or biological material

Policy information about studies with human participants or human data. See also policy information about sex, gender (identity/presentation), and sexual orientation and race, ethnicity and racism.

| | |
|---|---|
| Reporting on sex and gender | Not applicable |
| Reporting on race, ethnicity, or other socially relevant groupings | Not applicable |
| Population characteristics | Not applicable |
| Recruitment | Not applicable |
| Ethics oversight | Not applicable |

Note that full information on the approval of the study protocol must also be provided in the manuscript.

# Field-specific reporting

Please select the one below that is the best fit for your research. If you are not sure, read the appropriate sections before making your selection.

☒ Life sciences ☐ Behavioural & social sciences ☐ Ecological, evolutionary & environmental sciences

For a reference copy of the document with all sections, see nature.com/documents/nr-reporting-summary-flat.pdf

# Life sciences study design

All studies must disclose on these points even when the disclosure is negative.

| | |
|---|---|
| Sample size | No sample size calculations were performed. Sample sizes were chosen based on commonly accepted standards for the experimental methods used in the field. |
| Data exclusions | No samples were excluded except for outliers images in microscopy of condensates where images with out of focus (blurry) foci and images with signal too low to quantify accurately were excluded. |
| Replication | All conclusions presented in this study are supported by findings that were independently reproduced in biological replicate experiments. |
| Randomization | Not relevant |
| Blinding | Not relevant |

# Reporting for specific materials, systems and methods

We require information from authors about some types of materials, experimental systems and methods used in many studies. Here, indicate whether each material, system or method listed is relevant to your study. If you are not sure if a list item applies to your research, read the appropriate section before selecting a response.

## Materials & experimental systems

| n/a | Involved in the study | |
|---|---|---|
| ☐ | ☒ | Antibodies |
| ☐ | ☒ | Eukaryotic cell lines |
| ☒ | ☐ | Palaeontology and archaeology |
| ☒ | ☐ | Animals and other organisms |
| ☒ | ☐ | Clinical data |
| ☒ | ☐ | Dual use research of concern |
| ☒ | ☐ | Plants |

## Methods

| n/a | Involved in the study | |
|---|---|---|
| ☒ | ☐ | ChIP-seq |
| ☒ | ☐ | Flow cytometry |
| ☒ | ☐ | MRI-based neuroimaging |

# Antibodies

| | |
|---|---|
| Antibodies used | murine anti-MBP monoclonal antibody (E8032S, NEB)<br>goat anti-mouse IgG-HRP (AP308P, Chemicon)<br>mouse monoclonal anti-myc (2276S, Cell Signaling Technology)<br>goat anti-mouse IgG Alexa Fluor™ Plus 488 (A32723, Invitrogen)<br>mouse monoclonal anti-V5 (R96025, Invitrogen)<br>mouse monoclonal anti-PGK1 (ab113687, Abcam)<br>rabbit anti-phospho H2A-S129 (ab15083, abcam)<br>donkey anti-rabbit IgG Alexa Fluor™ 546 (A10040, Invitrogen) |
| Validation | These antibodies are standard reagents in molecular biology and their specificity for use in yeast is well established. Our study directly validates anti-MBP and anti-mouse antibody in Figure 5B, anti-myc and anti-mouse Alexa Fluor 488 antibody in Figures 3A, 3C, 4B where mutant shows reduced signal as compared to wild-type. Anti-V5 antibody is validated in Figure S6C where 0 h control shows no signal. Validation for for anti-phospho H2A-S129 antibody is available at https://doc.abcam.com/datasheets/active/ab15083/en-us/histone-h2a-phospho-s129-antibody-ab15083.pdf, for anti-PGK1 antibody at https://doc.abcam.com/datasheets/active/ab113687/en-us/pgk1-antibody-22c5d8-ab113687.pdf, and for anti-rabbit IgG Alexa Fluor™ 546 antibody at https://www.thermofisher.com/order/genome-database/dataSheetPdf?producttype=antibody&productsubtype=antibody_secondary&productId=A10040&version=Local. |

# Eukaryotic cell lines

Policy information about cell lines and Sex and Gender in Research

| | |
|---|---|
| Cell line source(s) | Sources of all yeast strains are given in Table S4. Spodoptera frugiperda Sf9 cells for expression of recombinant proteins were from Gibco (Thermo Fischer 11496015). |
| Authentication | Yeast strains were validated by PCR and/or Southern blotting. Sf9 certificates of analysis are available at https://assets.thermofisher.com/TFS-Assets/certificate/CM/COA/COA_11496015_03022023183049_2566577_1.pdf. |
| Mycoplasma contamination | Not available for yeast strains. Mycoplasma testing of Sf9 cells is referenced at https://assets.thermofisher.com/TFS-Assets/certificate/CM/COA/COA_11496015_03022023183049_2566577_1.pdf. |
| Commonly misidentified lines<br>(See ICLAC register) | Not available (yeast strains and Sf9 cells only) |

# Plants

| | |
|---|---|
| Seed stocks | *Report on the source of all seed stocks or other plant material used. If applicable, state the seed stock centre and catalogue number. If plant specimens were collected from the field, describe the collection location, date and sampling procedures.* |
| Novel plant genotypes | *Describe the methods by which all novel plant genotypes were produced. This includes those generated by transgenic approaches, gene editing, chemical/radiation-based mutagenesis and hybridization. For transgenic lines, describe the transformation method, the number of independent lines analyzed and the generation upon which experiments were performed. For gene-edited lines, describe the editor used, the endogenous sequence targeted for editing, the targeting guide RNA sequence (if applicable) and how the editor was applied.* |
| Authentication | *Describe any authentication procedures for each seed stock used or novel genotype generated. Describe any experiments used to assess the effect of a mutation and, where applicable, how potential secondary effects (e.g. second site T-DNA insertions, mosiacism, off-target gene editing) were examined.* |
